# Virtual Reality for Preoperative Planning and Education in Pediatric Surgery: Preliminary Results for the Treatment of Congenital Malformations and Tumors

**DOI:** 10.1002/wjs.12594

**Published:** 2025-04-17

**Authors:** Gloria Pelizzo, Ugo Maria Pierucci, Michela Marinaro, Sara Costanzo, Eleonora Durante, Carlotta Ardenghi, Alessia Musitelli, Paolo Milani, Francesco Rizzetto, Maurizio Vertemati, Alessandro Campari, Marta Barisella, Tommaso Santaniello, Cristina Gallotta, Anna Camporesi, Irene Paraboschi, Alessandro Varrica, Daniele Alberti, Valeria Calcaterra, Gianvincenzo Zuccotti

**Affiliations:** ^1^ Department of Pediatric Surgery “V. Buzzi” Children's Hospital Milano Italy; ^2^ Department of Biomedical and Clinical Science University of Milano Milano Italy; ^3^ CIMaINa (Interdisciplinary Centre for Nanostructured Materials and Interfaces) University of Milano Milano Italy; ^4^ Department of Physics “Aldo Pontremoli” University of Milano Milano Italy; ^5^ Department of Radiology ASST Grande Ospedale Metropolitano Niguarda Milano Italy; ^6^ Postgraduate School of Diagnostic and Interventional Radiology University of Milano Milano Italy; ^7^ Department of Pediatric Radiology “V. Buzzi” Children's Hospital Milan Italy; ^8^ Pathology Unit Sacco Hospital Milano Italy; ^9^ Department of Pediatric Anesthesia and Intensive Care “V. Buzzi” Children's Hospital Milano Italy; ^10^ Department of Congenital Cardiac Surgery IRCCS Policlinico San Donato San Donato Milanese Italy; ^11^ Department of Pediatric Surgery ASST Spedali Civili Children Hospital University of Brescia Brescia Italy; ^12^ Department of Clinical and Experimental Sciences University of Brescia Brescia Italy; ^13^ Pediatric Department “V. Buzzi” Children's Hospital Milano Italy; ^14^ Pediatrics and Adolescentology Unit Department of Internal Medicine University of Pavia Pavia Italy

**Keywords:** congenital malformations, education, pediatric surgery, preoperative simulation, tumors, virtual reality

## Abstract

**Purpose:**

Virtual reality (VR) has emerged as a valuable tool in surgical planning, offering detailed anatomical spatial orientation and three‐dimensional (3D) navigation. This study explores using virtual reality head‐mounted display (VR‐HMD) technology in preoperative planning for pediatric surgery, aiming to improve outcomes in treating congenital malformations and tumors while advancing surgical education.

**Methods:**

A retrospective analysis was performed on pediatric patients diagnosed with congenital malformations and tumors who received treatment between 2021 and 2024 at the “V. Buzzi” Children's Hospital in Milan, Italy. Patient‐specific 3D VR models were generated from reconstructed computed tomography/magnetic resonance imaging (CT/MRI) images and analyzed preoperatively to optimize surgical planning and strategy development. The advantages of preoperative VR compared to traditional imaging techniques were assessed.

**Results:**

Fifty VR models were included in the study (*n* = 35: congenital malformations and *n* = 15 tumors). The VR‐HMD setup facilitated interactive anatomical exploration, enabling precise surgical navigation and planning. Compared to conventional imaging, preoperative VR simulations modified the surgical approach in 75.0% of cases, enabling minimally invasive strategies in complex congenital malformations and guiding the decision for open surgery in anatomically challenging tumors such as adrenal and hepatic masses. VR images demonstrated superior anatomical resolution and identified potential intraoperative complications in 92.0% of cases compared to conventional imaging. Remarkably, examining the vascular hilum in pulmonary, hepatic, and renal structures provided enhanced guidance for determining the surgical approach, ensuring a safer and more precise respect for the anatomy in complex cases. As a result, preoperative VR navigation demonstrated the feasibility of minimally invasive procedures in 45.6% of complex cases whereas recommending an open surgical approach in 55.4% of the models. Limitations in visualizing urological structures (e.g., ureters and bladder in complex urogenital malformations) limited the VR's utility in those cases, underscoring the need for future advancements in segmentation and multimodal imaging to enhance anatomical accuracy.

**Conclusion:**

Preoperative VR enables customized surgical planning, potentially minimizes intraoperative risks, and provides valuable educational opportunities for pediatric surgical teams. Future advancements in VR technology promise to further enhance its integration into clinical practice, ultimately improving pediatric patients' outcomes.

## Graphical abstract

Virtual reality (VR) has emerged as a valuable tool in pediatric surgical planning, offering detailed anatomical spatial orientation and three‐dimensional (3D) navigation.

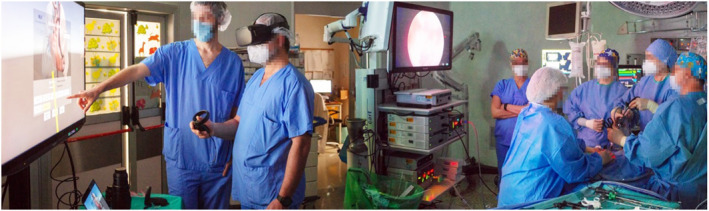

**Abbreviations**
AR: Augmented reality
CLM: Congenital lung malformations
CT: Computed tomography
DICOM: Digital Imaging and Communications in Medicine
EU: European Union
EUPSA: European Association of Pediatric Surgeons
HMD: Head‐mounted display
MIS: Minimally invasive surgery
MR: Mixed reality
MRI: Magnetic resonance imaging
MUSA: Multilayered Urban Sustainability Action
NRRP: National Recovery and Resilience Plan
OR: Operating room
VATS: Video‐assisted thoracoscopic surgery
VR: Virtual reality
VRE: Virtual reality environment
WHO: World Health Organization
3D: Three‐dimensional

## 1 Introduction

Pediatric surgery is a highly specialized surgical discipline that requires technical expertise in treating complex congenital malformations and pediatric tumors from infancy to adolescence [1, 2]. The unique understanding of the anatomical development in children, which progresses quantitatively and qualitatively compared to adults, sets this field apart from other surgical specialties as a reconstructive rather than destructive surgery [3]. The success of reconstructive surgery for malformations and tumors depends mainly on the surgeon's ability to understand anatomical variants [4, 5]. Therefore, a complete preoperative understanding of the anatomy is essential to reduce errors and improve surgical outcomes [5, 6, 7, 8].

International guidelines and national healthcare regulations highlight the significance of simulation for the ongoing training needed by experienced and younger surgeons and the entire medical team as a standard for delivering quality healthcare [9, 10, 11]. The World Health Organization (WHO) and the European Association of Pediatric Surgeons (EUPSA) recommend using advanced technologies to support clinical practice, which can improve skills, reduce errors, enhance outcomes, and minimize surgical invasiveness, hospital stays, pain, and esthetic complications [12].

Virtual reality head‐mounted display (VR‐HMD) technology has been introduced to achieve these goals in clinical practice. This technology helps with anatomical spatial orientation and three‐dimensional (3D) preoperative navigation of an anatomical district through a patient‐specific orientation. In particular, VR platforms and immersive technologies enable the examination of a patient's anatomical complexity outside the operating room (OR), improving information retention and recall, reducing memory effort, and lowering surgeon stress [4, 5].

Recent studies have shown that VR applied to adult patient surgery increases surgeon safety by over 60 times, changes the decision‐making process in 10% of cases, and reduces the incidence of intraoperative errors in 40% of cases [13, 14].

Recently, VR‐HMD technologies have also been introduced in pediatric surgery to aid in preoperative surgical planning and anticipate potential surgical difficulties in reconstructive surgery. However, their application remains limited, and only a few studies have explored their role in the pediatric population, particularly in the context of congenital malformations and tumors [4, 5, 8]. Compared to adult surgical disciplines, where the clinical benefits of VR are increasingly well‐documented, the use of VR in pediatric surgery is still in its early stages. This is especially true for complex malformations, where anatomical variability and lower‐case volumes pose unique challenges. In our experience, we have cited the only available studies found in the literature, underlining our approach's novelty and potential impact. This study aims to evaluate the role of VR‐HMD technology in preoperative planning and surgical education for pediatric patients affected by congenital malformations and tumors. We aimed to determine whether the integration of VR into the surgical workflow could modify the planned approach compared to traditional imaging alone, enhance anatomical understanding, and support decision‐making and training within the multidisciplinary surgical team.

## 2 Materials and Methods

### 2.1 Patients

A retrospective analysis was conducted from 2021 to 2024, including all patients affected by congenital malformations and tumors who underwent surgery at the Department of Pediatric Surgery at “V. Buzzi” Children's Hospital in Milan, Italy, for whom a VR HMD was set up to plan a preoperative surgical strategy.

Demographic, clinical, and preoperative data, intraoperative findings, outcomes, and postoperative feedback were documented. The 3D‐reconstructed computed tomography/magnetic resonance imaging (CT/MRI) images were preoperatively evaluated in each patient. An analysis of anatomic details from the 3D VR imaging was conducted for each case. Preoperative navigation using VR and VR‐HMD technologies encompassed all anatomical abnormalities, facilitating the planning of surgical strategies. The surgical strategy developed using VR was compared to the steps planned with traditional imaging techniques. Additionally, the study examined whether the surgical approach was influenced by the review of 3D VR imaging and assessed the alignment between 3D reconstructions and intraoperative findings observed using VR. The study was conducted following the permission of the local Ethical Committee (N 2022/ST/079).

### 2.2 Three‐Dimensional (3D) Models

To create a highly detailed 3D model of a patient's malformation or tumor, a CT scan with a slice thickness of 0.6 mm was performed during both the arterial and venous phases, accounting for the small size of anatomical structures in pediatric patients. The resulting CT images were exported as DICOM files and processed using 3D Slicer v4.11, a free and open‐source software designed for advanced medical image analysis [15]. The software enabled precise image segmentation, which involves labeling anatomical structures in medical images to distinguish them from the background and one another.

Due to the complexity of the structures, a semiautomatic segmentation method was employed. This method integrated built‐in region‐growing and threshold algorithms with manual refinements to ensure accuracy. The segmented data were then used to create 3D surface models of the malformation and surrounding anatomical structures. These models allowed for detailed inspection, with features such as zooming, multiangle viewing, and the ability to hide or display individual models or apply transparency. Unlike traditional volume‐rendering techniques, this approach made it easier to visualize internal vascularization or isolate specific structures for analysis.

Finally, a radiologist reviewed and approved the segmentations and the resulting 3D scenes for consistency with the original medical data images.

### 2.3 Virtual Reality Head‐Mounted Display (VR‐HMD) Model

We used a custom‐developed plugin for 3D Slicer to import 3D models of a patient's malformation or tumor into an Oculus Quest v.1 (META Inc., Menlo Park, CA, USA) virtual reality (VR) headset. The headset has an OLED display with a resolution of 1440 × 1600 pixels per eye and a refresh rate of 72 Hz.

A previously developed application for the Oculus Quest was adapted to enable the surgical team to visualize the 3D reconstructions within a dedicated virtual reality environment (VRE).

**FIGURE 1:**
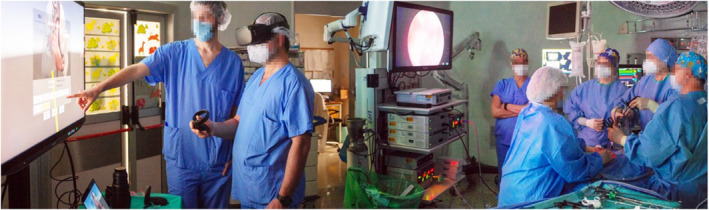
Integration of virtual reality (VR) technology into the surgical workflow. The surgical team uses VR head‐mounted displays (HMDs) and interactive screens for preoperative planning and intraoperative guidance, enhancing precision and collaboration during complex procedures.

During preoperative planning, surgeons wore the VR headset and used its wireless controllers to launch the app, choose a reconstruction, and interact with it. They could manipulate the models by moving, rotating, zooming, and adjusting transparency to focus on specific anatomical features. A connected computer running a dedicated desktop application allowed the surgeon's perspective to be shared with the rest of the team, facilitating discussion and agreement on the best surgical approach (Figure 1). A comprehensive and interactive VR exploration of the anatomical representation was performed preoperatively, with the images obtained compared to traditional CT/MRI data. The surgical approach was determined after carefully evaluating the orientation of critical structures and achieving a thorough understanding of complex anatomy. Focus was placed on analyzing the vascular systems and parenchymal structures of the liver, kidneys, and lungs vascular systems and parenchymal anatomy as well as the biliary system's anatomy and spatial relationship with the hepatic vasculature.

### 2.4 Preoperative Virtual Reality Head‐Mounted Display (VR‐HMD) Setup Evaluation

The preoperative evaluation was conducted using CT images to plan surgeries, utilizing patient‐specific 3D models visualized in the VRE. Preoperative navigation through critical anatomical structures, including vessels, the biliary tract, and ureters, was performed by rotating and orienting the images to replicate the patient's positioning on the operating table. The VR‐based navigation faithfully mirrored the patient's actual intraoperative positioning, enabling precise planning of all surgical steps, from the initial incision or trocar placement (for minimally invasive procedures) to subsequent stages, such as vessel dissection, ligation, and parenchymal resection. Patient‐specific preoperative simulations were carried out with the surgical team a few days before surgery and immediately during the preoperative period. Postoperatively, all surgeons were asked to evaluate and list the advantages of preoperative VR.

Preoperative navigation findings were compared to traditional imaging and conventional 3D reconstructions to validate their accuracy and clinical relevance. The surgical team's feedback focused on key factors. A simplified scoring system was retrospectively applied to each surgical procedure based on feedback from the operating surgeons. Four key technical steps were evaluated to determine the perceived benefit of VR navigation: (1) choice of incision or trocar placement before and after virtual navigation; (2) facilitation in identifying anatomical structures; (3) dissection and ligation of vascular structures; and (4) resection or excision of the anatomical lesion. A score of 1 was assigned when at least three of these four steps were judged to have been positively influenced using VR; a score of 0 was applied when fewer than three showed perceived improvement. The surgical team discussed and agreed upon this evaluation following the procedure.

### 2.5 Surgical Technique

The VR workstation and the 3D reconstruction, displayed on a monitor, were situated in the OR to offer extra anatomical review during surgery. Skilled pediatric surgeons performed thoracic or abdominal procedures, depending on the surgical requirements. Two surgical approaches were employed: minimally invasive procedures, including laparoscopic or thoracoscopic techniques, and open surgeries, such as laparotomy or thoracotomy. When necessary, interventional radiological procedures were incorporated during the operation.

## 3 Results

A total of 50 VR models were created to assist in preoperative planning. The median patient age at surgery was 7 years (range: 2 months to 18 years). Thirty‐five models were developed for various congenital malformations (*n* = 19 pulmonary, *n* = 9 biliopancreatic, *n* = 4 urogenital, *n* = 2 splenic, and *n* = 1 intestinal). Additionally, 15 models were constructed for neoplastic diseases: 7 neuroblastomas, 2 lung tumors, 2 hepatic tumors, and 4 renal tumors (Table 1). Pulmonary lesions represented the most significant subset, comprising 54.3% of all congenital cases. The preoperative evaluation of these models enabled the surgical team to determine the optimal approach for addressing the complex anatomical variations unique to each patient. A direct correlation was observed between the preoperative VR HMD findings and the intraoperative findings in all cases.

**TABLE 1:** Overview of VR preoperative planning.

Type of lesion	Cases (*n*)
Congenital malformations	35
Pulmonary	19
Biliopancreatic	9
Urogenital	4
Splenic cysts	2
Intestinal	1
Neoplastic diseases	15
Neuroblastomas	7
Pulmonary tumors	2
Hepatic tumors	2
Renal tumors	4
Total	50

In 92% of cases (46 out of 50), VR provided superior anatomical resolution compared to CT/MRI imaging, allowing surgeons to identify 3D organ‐blood vessel relationships and anticipate potential vascular incidents and surgical complications. In 4 out of 50 cases (8.6%), VR did not offer additional benefits over traditional imaging. These were mainly urogenital malformations where the segmentation of the urinary tract (e.g., ureters and bladder) was incomplete or not sufficiently accurate for effective preoperative planning. In these cases, standard imaging remained the primary reference for surgical strategy. However, the detailed anatomical visualization afforded using VR navigation influenced surgical planning in 75.0% of cases, significantly modifying the approach compared to evaluations based solely on conventional imaging. Notably, VR allowed surgeons to simulate surgical maneuvers, identifying potential risks such as capsule rupture in adrenal tumors, vascular injuries in the lungs, and the feasibility of renal resection instead of nephrectomy. The choice of open rather than minimally invasive procedure was based on the criteria of advantages and disadvantages detected in preoperative VR.

In summary, the MIS was performed when all the criteria (i.e., spatial understanding, surgical precision, time efficiency, and enhanced confidence) were present. Open surgery was chosen when the time efficiency and enhanced confidence criteria were prevalent. These improvements enabled a safer and more personalized surgical strategy, contributing to the concept of “Tailored Surgery” (Table 2). In several cases, VR contributed to modifying the surgical plan. For instance, VR planning facilitated a thoracoscopic approach in congenital lung malformations where segmental bronchial anatomy was better appreciated (18/19 cases, 95%). Conversely, in the adrenal (3/4 cases, 75%) and hepatic tumors (2/2 cases, 100%), where the VR model revealed proximity to major vessels, the team opted for open surgery to ensure vascular control.

**TABLE 2:** Comparison of VR navigation advantages, traditional imaging benefits, and surgical approaches (minimally invasive vs. open surgery) across different lesion types and cases treated at our center.

Lesion type	Total cases (*n*)	VR navigation advantages	Traditional imaging advantages	Minimally invasive surgery	Open surgery
Pulmonary malformations	19	19/19 (100%)	0/19 (0%)	18/19 (95%)	1/19 (5%)
Biliopancreatic malformations	9	9/9 (100%)	0/9 (0%)	0/9 (0%)	9/9 (100%)
Urogenital malformations	4	0/4 (0%)	4/4 (100%)	1/4 (25%)	3/4 (75%)
Splenic cysts	2	2/2 (100%)	0/2 (0%)	2/2 (100%)	0/2 (0%)
Intestinal malformations	1	1/1 (100%)	0/1 (0%)	0/1 (0%)	1/1 (100%)
Neuroblastomas	7	6/7 (86%)	1/7 (14%)	3/7 (43%)	4/7 (57%)
Pulmonary tumors	2	2/2 (100%)	0/2 (0%)	0/2 (0%)	2/2 (100%)
Hepatic tumors	2	2/2 (100%)	0/2 (0%)	0/2 (0%)	2/2 (100%)
Renal tumors	4	3/4 (75%)	1/4 (25%)	1/4 (25%)	3/4 (75%)
Overall	50	46/50 (91.4%)	4/50 (8.6%)	22/50 (45.6%)	28/50 (55.4%)

*Note:* Advantages include accuracy and clinical relevance (score = 1 almost 3 of the following 4 characteristics are reported: spatial understanding, surgical precision, time efficiency, and enhanced confidence and score 0 includes only 1 of the criteria).

### 3.1 Thoracic Surgery

**FIGURE 2:**
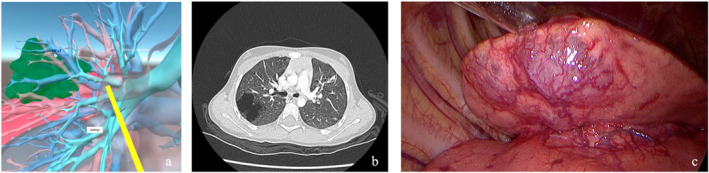
Multimodal imaging and intraoperative views of congenital pulmonary airway malformation (CPAM). (a) VR reconstructed anatomical visualization of CPAM, highlighting airway anomalies with a yellow marker indicating the segmental region of interest; (b) Axial CT scans illustrating the abnormal pulmonary architecture and presence of a cystic lesion consistent with CPAM; (c) Intraoperative photo depicting the gross appearance of the malformation during surgical resection, showcasing vascularity and cystic structure.

**FIGURE 3:**
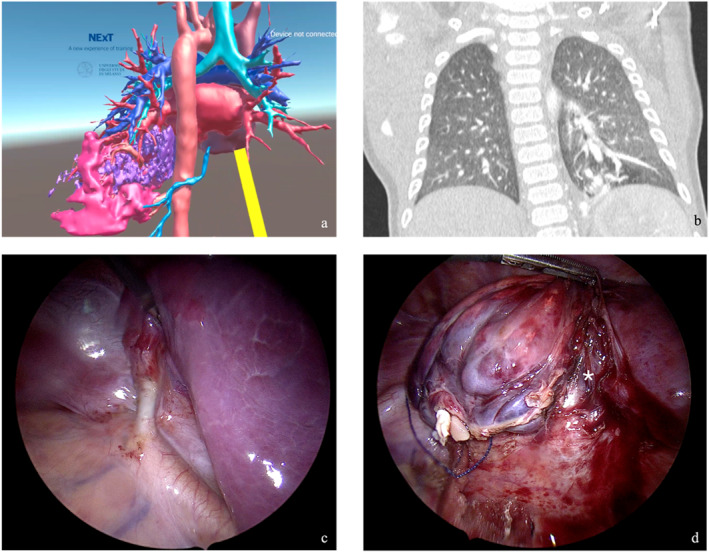
Multimodal imaging and intraoperative views of an intralobar sequestration: (a) Virtual reality (VR) reconstruction showing intralobar sequestration with vascular hyperafflux (highlighted in pink) and associated cystic malformation (highlighted in purple). (b) CT scan illustrating the pulmonary malformation with abnormal vascularization and cystic architecture. (c) Intraoperative view of the afferent arterial vessel from the aorta supplying the pulmonary sequestration. (d) Intraoperative view during a left lower pulmonary lobectomy; isolation of the lower bronchus (*) and ligation of vascular structures.

Preoperative VR navigation of the pulmonary hilum provided precise anatomical visualization, greatly enhancing the understanding of the hilum's complex vascular and bronchial variations. This approach allowed surgeons to replicate the preoperative navigation methods even during surgery, facilitating a more accurate intraoperative dissection. Careful consideration of preoperative VR navigation was essential for optimizing patient positioning. VR technology improved planning for surgical approaches, trocar placement in minimally invasive procedures, and incision design in open surgeries. In the case of lung tumors, VR evaluation offered a more detailed overview of the hilum compared to conventional radiology (Figures 2 and 3). In two cases, the VR preoperative study was the only method that revealed the involvement of the hilum within the pericardial space. The surgical plan was changed, and a sternotomy was carried out to allow an intrapericardial approach to the pulmonary hilum.

The VR preoperative navigation helped confirm the approach's feasibility (minimally invasive or open). Based on the criteria above—spatial understanding, surgical precision, time efficiency, and enhanced confidence—thoracoscopy proved to be the most suitable procedure for treating complex congenital malformations (Table 1). However, VR‐HMD revealed limitations in cases requiring pulmonary parenchyma resection and subsegmental bronchial dissection. For these patients, open surgery was deemed the technique of choice.

### 3.2 Gastrointestinal Surgery

Although laparoscopic approaches were initially deemed feasible for liver and biliopancreatic surgeries based on standard imaging, VR‐HMD evaluations uncovered significant anatomical constraints that ruled out the suitability of minimally invasive techniques. This change in surgical strategy emphasized the vital role of VR in preoperative planning, especially for complex hepatobiliary cases. VR provides detailed insights into critical anatomical relationships and identifies potential complications that could be missed with conventional imaging techniques.

### 3.3 Urology

VR models displayed distinct advantages in nephron‐sparing surgeries by providing detailed visualization of renal anatomy that was not achievable with conventional CT imaging (Table 3). However, for malformations involving the renal collecting system, ureters, uterus, or vagina, VR visualization required further methodological refinements to achieve the accuracy of traditional radiological techniques. In these cases, conventional imaging played a more critical role in guiding surgical decisions.

**TABLE 3:** Overview of VR advantages and limitations in preoperative planning across various anatomical structures.

Anatomical structures	VR advantages	VR limitations
Lung	Detection of anatomical variants	Limited control in tissue resection
	High precision in vessel orientation relative to parenchyma	Limited benefit for subsegmental bronchial dissection
	High accuracy in deep spatial perception	
Liver and biliary tree	Very high precision in identifying vascular supply	Limited control in resection of duodenal and pancreatic tissues
Kidney and urogenital system	High accuracy in identifying vascular anomalies of the parenchyma	Limited accuracy for vascular supply to calyces, pelvis, ureters, and bladder
Adrenal lesions	High precision in visualizing adrenal veins and arteries	Risk of underestimating capsular rupture in hormonally active adrenal adenomas
	Detailed spatial understanding of adrenal gland relationships with surrounding vascular and parenchymal tissues	Limited benefit for evaluating small or hormonally active lesions where VR can miss capsular fragility

### 3.4 Oncology

**FIGURE 4:**
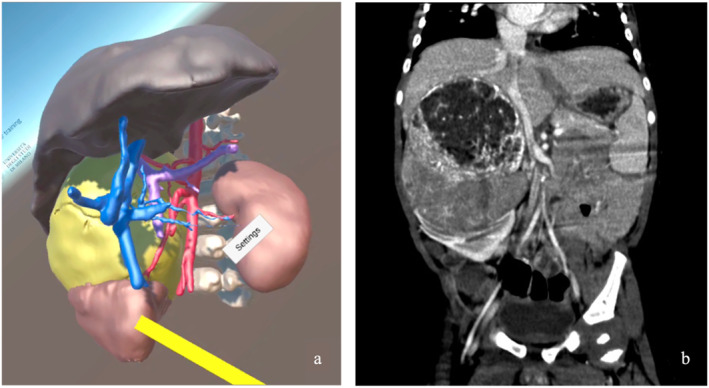
Multimodal representation of the right adrenal neuroblastoma: (a) VR reconstructed anatomical visualization showing the tumor's spatial relationship with adjacent organs and artery distribution, highlighted with a yellow marker and (b) coronal CT scan demonstrating the large abdominal mass with heterogeneous density, indicative of its complex nature and involvement with surrounding tissues.

Preoperative VR assessments proved particularly valuable for intraparenchymal tumors, such as hepatoblastomas and pulmonary lesions. The VR models provided detailed insights into the proximity of tumors to critical vascular structures, including hepatic and pulmonary veins and portal anatomy, significantly enhancing surgical planning (Figure 4). VR‐HMD proved highly effective in vascular mapping for neuroblastomas and Wilms' tumors, enabling precise planning for partial nephrectomy or tumor excision. VR's 3D visualization capabilities offered unparalleled precision in assessing deep vascular structures.

At the same time, although video‐assisted thoracoscopic surgery (VATS) procedures demonstrated strengths in tissue resection, they lacked the spatial depth provided using VR models. However, a limitation emerged in the system's ability to evaluate tissue consistency and fragility, as in hormonally active adrenal adenomas, where the risk of capsular rupture was underestimated. This is not a limitation of the VR interface itself but instead of the current imaging, segmentation, and modeling process, which cannot encode biomechanical or functional properties of tissues. Future developments may include the integration of radiomics, elastography, diffusion‐weighted imaging, or machine learning‐based classifiers to enrich 3D models with data on tissue density, stiffness, or fragility [16, 17]. Such advancements could substantially enhance the accuracy and predictive value of VR‐based simulations for preoperative planning.

## 4 Discussion

VR in medicine has gained significant traction in recent years, particularly in surgery, where it has enhanced intraoperative orientation, surgical outcomes, and training [18]. Studies in adult patients demonstrate VR's ability to boost surgeon confidence over 60 times and influence decision‐making in approximately 10% of cases [13, 19]. However, the integration of VR for preoperative planning, a relatively recent breakthrough introduced in 2017, has primarily focused on adult populations, especially in neurosurgical and urological fields [20, 21].

Though promising, VR's application in pediatric surgery remains underexplored. Limited literature exists on the use of VR‐HMD technologies in pediatric patients. The most comprehensive study to date, involving 400 pediatric cases across U.S. institutions in 2023, highlighted VR's prominent role in pediatric oncology and cardiac surgery [6, 22].

Our retrospective analysis of 50 VR models in pediatric patients supports prior findings, showing that VR significantly enhances anatomical orientation and surgical planning. Notably, VR influenced the surgical approach in 75.0% of cases in our study, aligning with the reported 65% enhancement in surgical precision achieved using preoperative 3D models [23, 24]. Additionally, our findings corroborate with Brown et al. [25] demonstrating VR's capacity to identify potential vascular incidents overlooked using traditional radiology. These results underline VR's potential to provide a more detailed and comprehensive understanding of critical anatomical relationships.

Our study highlights VR's ability to facilitate less invasive and more precise surgical approaches, particularly in thoracic surgery. For instance, VR modeling enabled decisions favoring limited resections over standard lobectomies in congenital lung malformation (CLM) cases. This advantage is particularly valuable for pediatric patients, where minimizing invasiveness can lead to better outcomes. However, challenges persist in specific anatomical regions, such as the ureteral and pelvic collector systems, where current VR technology struggles with accurate visualization (Table 3). These limitations necessitate further technological advancements to optimize VR's efficacy in these areas.

## 5 Innovative Aspects

### 5.1 Preoperative Planning and Training

Our experience began with CLM in thoracic pediatric surgery and has since grown to encompass other pediatric surgical specialties, such as abdominal and urogenital surgery.

Due to each congenital case's unique and varied orientation, CLM represented an ideal substrate for VR's impact [5]. Other medical centers have reported similar experiences and benefits in cardiothoracic surgery, and there has been an increased demand for these procedures over time [26, 27]. Our initial experience in thoracic surgery provided valuable insights into VR technology's impact, shaping and guiding our focus for future applications and efforts in other surgical specialties.

### 5.2 Simulation and Skilling

Preoperative navigation is crucial because, unlike traditional cross‐sectional imaging, VR preoperative navigation enabled clinicians to interactively explore the patient's anatomy by rotating, zooming, and resizing images. This helped them gain a better understanding of the anatomical complexity and the surrounding tissues. The VR models enabled all surgeons to immerse themselves in and better understand the complex anatomical structures surrounding the surgical site. In other words, the VR models allowed surgeons to virtually step inside the malformation or tumor, enabling them to explore the surgical path from within rather than merely observing it from an external perspective. The typical report from the experience of other centers is a recurrent comment of the surgeons who felt like they “had been there before” [8]. This capability stems from the unique features of the VR system, particularly its application in preoperative planning, which proves to be an invaluable tool for deciphering complex anatomical structures. Understanding the anatomy requires a mental integration of a preoperatively constructed mental model derived from traditional radiology (i.e., 2D imaging) and intraoperative anatomical findings. From a practical point of view, the VR system offers multiple advantages (Table 3). For example, it reduced surgeons' anxiety and enabled a rapid learning curve for young surgeons. Moreover, VR platforms and immersive technologies allowed for exploring the patient's anatomical complexity outside the OR. This enhanced information retention and recall, minimized memory load, and reduced surgeon stress. The mental effort required to comprehend complex anatomy was significantly decreased when using VR procedures [28].

As reported by Robertson et al. [8], a subjective increase in confidence was reported in 63% of the cases and the operation planning changed in 8.3% of instances.

VR also holds significant potential for clinician training and simulation, providing opportunities to gain and enhance experience outside direct patient care. For residents, VR simulation complements traditional simulators by replicating clinical scenarios in thoracic, abdominal, and urogenital fields, improving the training experience. Moreover, 3D VR models can facilitate team‐based surgical discussions, strengthen collaboration in addressing surgical challenges, and provide technical training while exploring potential solutions for complex cases.

## 6 Patient Care and Education, Sharing Care, and Therapeutic Alliance

Patient education is a part of informed consent. In complex malformations, explaining intricate 3D anatomy can be challenging for surgeons, whereas parents may struggle to grasp the condition's complexity and implications for their child. 3D models created through VR can significantly improve communication, facilitating shared decision‐making between surgeons (e.g., surgeons worldwide could use cloud‐based VR platforms to plan surgeries in real time and integrate their expertise collaboratively) and patients' families (e.g., VR could be used to explain surgical procedures to patients and their families in a comprehensible way, reducing anxiety and improving consent processes).

We are currently collecting survey data at our two separate centers regarding patient/parent experience when the VR model is used as a part of the preoperative education and informed consent process.

Emerging perspectives on using VR in clinical practice offer the potential for precise delineation of lesions and major anatomical details, paving the way for a new model of understanding and applying anatomy in surgical planning and education. Moreover, this knowledge is strictly addressed to a tailored surgery dedicated to a single patient as a unique model.

## 7 Study Design Limitations

This study has several limitations. Its retrospective design and absence of a control group limit the ability to draw direct comparisons between VR and traditional imaging. The sample size, although among the largest pediatric VR experiences reported to date, remains relatively small. In cases of urological malformations, standard imaging was used alongside VR due to segmentation limitations.

### 7.1 Current Limitations of VR and Future Perspectives

Consistent with findings from previous studies, our work acknowledges certain limitations in using VR, particularly in visualizing the ureteral and renal collecting systems. Current VR technology remains challenged in these areas, necessitating future advancements to enhance accuracy and effectiveness. Future research should prioritize refining VR capabilities in these critical areas and exploring further integration of VR into pediatric surgical practice.

A current limitation of this study is the lack of real‐time intraoperative guidance, such as the ability to overlay virtual anatomical models onto the surgical field. However, this is not a technical limitation of VR but rather a feature associated with augmented reality (AR) or mixed reality (MR) technologies specifically designed to integrate digital content with the physical environment [29, 30]. Future research may explore incorporating AR into surgical navigation systems to allow dynamic superimposition of virtual models on real anatomical structures, potentially enhancing surgical precision during live procedures. Hybrid devices capable of supporting both VR and AR modalities may offer novel avenues for developing integrated preoperative‐to‐intraoperative workflows. Our study supports and expands the existing literature on the role of VR in surgery, demonstrating its specific value in the planning and execution of pediatric surgical procedures. Although further technological refinements are needed, VR stands out as a promising tool for reducing operative risks and improving clinical outcomes in pediatric patients. Preoperative VR serves as a training and simulation tool to reduce intraoperative minimally invasive and open errors, providing surgical team and trainees with realistic simulations of rare and complex pediatric conditions. These simulations would allow for unlimited practice in a risk‐free environment. The application of VR in pediatric surgery strengthens technological innovation as a fundamental tool for quality control and performance monitoring, both at a managerial and professional level. Moreover, VR models could also be used to create a repository of anonymized VR‐based reconstructions of past cases, serving as an invaluable resource for training and preparation. Investment in these cutting‐edge technologies can be strategic in ensuring an excellent healthcare service in line with the latest international guidelines and in providing the specific care needed for pediatric patients. Another consideration traditionally associated with VR technology is the potential for user discomfort, including cybersickness symptoms during prolonged or motion‐intensive sessions [31]. In our study, this risk was mitigated by the limited use of the VR headset: navigation sessions were brief and focused and video recordings of the VR simulations were used to support intraoperative discussions without requiring repeated immersion. Although no discomfort was reported, this remains a factor to be monitored in future applications involving extended or more interactive VR use.

## 8 Conclusions

VR and patient‐specific preoperative simulation support customized surgical planning and enrich educational opportunities for pediatric surgical teams. In our preliminary experience, VR improved anatomical understanding and influenced surgical decision‐making, especially in complex congenital malformations and tumors. Nonetheless, certain limitations were encountered, including segmentation challenges in urological anatomy and the retrospective design without a control group. These underscore the need for further validation. Future advancements in extended reality technologies, particularly the integration of AR systems capable of overlaying 3D models onto dynamic anatomical structures during surgery, may further enhance the intraoperative applicability of patient‐specific simulations. The combined use of VR for immersive preoperative planning and AR for real‐time surgical navigation could represent a powerful paradigm shift in pediatric surgical practice. As these technologies evolve, VR and preoperative simulation can revolutionize surgical approaches in pediatric patients, advancing toward a global standard of care that prioritizes safety, efficacy, and minimally invasive techniques.

## Author Contributions

**Gloria Pelizzo:** conceptualization, writing – original draft, supervision, methodology, validation, writing – review and editing, project administration, investigation. **Ugo Maria Pierucci:** methodology, investigation, validation, software, formal analysis, writing – original draft, writing – review and editing, data curation. **Michela Marinaro:** investigation, writing – original draft, data curation. **Sara Costanzo:** investigation, supervision. **Eleonora Durante:** investigation. **Carlotta Ardenghi:** investigation. **Alessia Musitelli:** investigation. **Paolo Milani:** investigation, methodology, software. **Francesco Rizzetto:** investigation, methodology, software. **Maurizio Vertemati:** investigation, methodology, software. **Alessandro Campari:** investigation, methodology, software. **Marta Barisella:** investigation. **Tommaso Santaniello:** investigation, software. **Cristina Gallotta:** investigation, software. **Anna Camporesi:** investigation. **Irene Paraboschi:** investigation, writing – review and editing, writing – original draft. **Alessandro Varrica:** investigation. **Daniele Alberti:** investigation. **Valeria Calcaterra:** validation. **Gianvincenzo Zuccotti:** supervision, validation, visualization.

## Ethics Statement

The data were retrospectively evaluated according to the local Ethical Committee N 2022/ST/079 principles (“VR and 3D models for simulation and research in pediatrics”).

## Consent

Informed written consent was obtained from the parents and/or legal guardian after receiving information about the study.

## Conflicts of Interest

The authors declare no conflicts of interest.

## Data Availability

Data reported in this study are available from the corresponding author upon request.
